# Ion-pumping microbial rhodopsins

**DOI:** 10.3389/fmolb.2015.00052

**Published:** 2015-09-22

**Authors:** Hideki Kandori

**Affiliations:** Department of Frontier Materials and OptoBioTechnology Research Center, Nagoya Institute of TechnologyNagoya, Japan

**Keywords:** light-driven pump, retinal, photoisomerizatoin, photocycle, H^+^ transfer, hydrogen bond, structural change

## Abstract

Rhodopsins are light-sensing proteins used in optogenetics. The word “rhodopsin” originates from the Greek words “rhodo” and “opsis,” indicating rose and sight, respectively. Although the classical meaning of rhodopsin is the red-colored pigment in our eyes, the modern meaning of rhodopsin encompasses photoactive proteins containing a retinal chromophore in animals and microbes. Animal and microbial rhodopsins possess 11-*cis* and all-*trans* retinal, respectively, to capture light in seven transmembrane α-helices, and photoisomerizations into all-*trans* and 13-*cis* forms, respectively, initiate each function. Ion-transporting proteins can be found in microbial rhodopsins, such as light-gated channels and light-driven pumps, which are the main tools in optogenetics. Light-driven pumps, such as archaeal H^+^ pump bacteriorhodopsin (BR) and Cl^−^ pump halorhodopsin (HR), were discovered in the 1970s, and their mechanism has been extensively studied. On the other hand, different kinds of H^+^ and Cl^−^ pumps have been found in marine bacteria, such as proteorhodopsin (PR) and *Fulvimarina pelagi* rhodopsin (FR), respectively. In addition, a light-driven Na^+^ pump was found, *Krokinobacter eikastus* rhodopsin 2 (KR2). These light-driven ion-pumping microbial rhodopsins are classified as DTD, TSA, DTE, NTQ, and NDQ rhodopsins for BR, HR, PR, FR, and KR2, respectively. Recent understanding of ion-pumping microbial rhodopsins is reviewed in this paper.

## Rhodopsins and light-driven ion pumps

**Figure 1:**
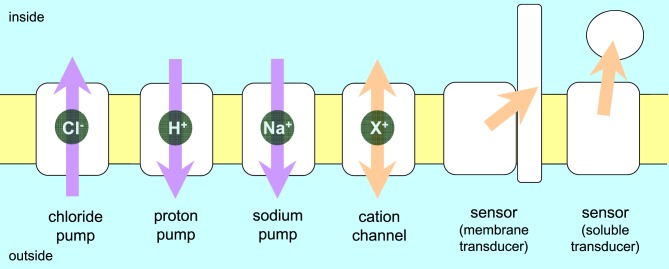
**Function of rhodopsins**. Animal rhodopsins are G-protein coupled receptors, which are categorized as sensors activating a soluble transducer. On the other hand, microbial rhodopsins can act as pumps, channel, and light-sensors. Arrows indicate the direction of transport or flow of a signal. Purple and orange arrows represent energy conversion and signal transduction, respectively.

The word “rhodopsin” originates from the Greek words “rhodo” and “opsis,” which indicate rose and sight, respectively. Thus, the classical meaning of rhodopsin is the red-colored pigment in the retinal rods of eyes. The chromophore molecule to absorb light is retinal, which is the origin of red color. Then, similar colored retinal-binding proteins were found in microbes, largely expanding the definition of the word rhodopsin. The modern meaning of rhodopsin encompasses photoactive proteins containing a retinal chromophore in animals and microbes (Ernst et al., 2014; Kandori, 2015). Rhodopsins are now found in all domains of life and are classified into two groups, animal and microbial rhodopsins. While animal rhodopsins are exclusively photosensory receptors as a specialized subset of G-protein coupled receptors, microbial rhodopsins have various functions, including as photosensory receptors, a light-switch for gene expression, photoactivatable enzymes, light-driven ion pumps and light-gated ion channels (Figure 1) (Ernst et al., 2014).

**Figure 2:**
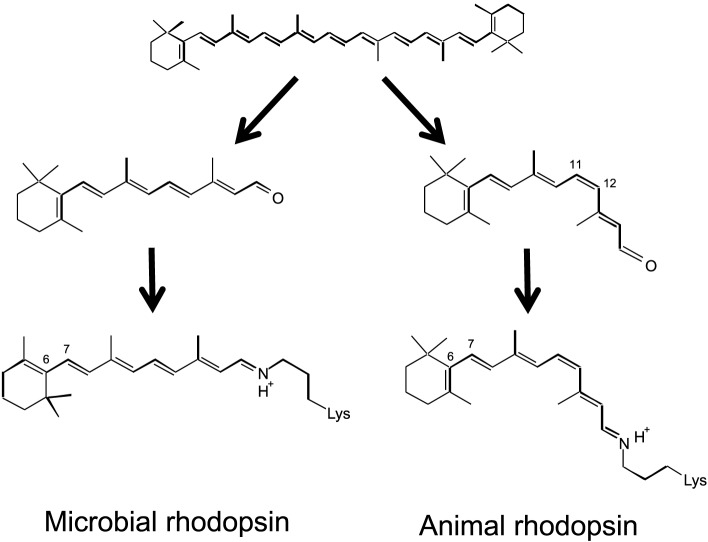
**Chromophore molecules of microbial (left) and animal (right) rhodopsins**. β-carotene (top) is the source of the chromophore, and all-*trans* and 11-*cis* retinal is bound to protein through the Schiff base linkage.

Microbial and animal rhodopsins share a common architecture of seven transmembrane α-helices with the N- and C-termini located extracellularly and intracellularly, respectively, but have almost no sequence homology and differ largely in their functions. Retinal, the aldehyde of vitamin A, is derived from β-carotene and is bound to the protein in the shape of all-*trans* and 11-*cis* forms in microbial and animal rhodopsins, respectively (Figure 2). Retinal is attached by a Schiff base linkage to the ε-amino group of a Lysine side chain in the middle of the 7th helix, and the retinal Schiff base is protonated in most cases (Figure 2).

Microbial rhodopsins were first found in the Archaea (*Halobacterium salinarum*) (Oesterhelt and Stoeckenius, 1971) and were therefore initially termed archaeal rhodopsins. *H. salinarum* contains bacteriorhodopsin (BR) (Oesterhelt and Stoeckenius, 1971) and halorhodopsin (HR) (Matsuno-Yagi and Mukohata, 1977; Schobert and Lanyi, 1982) that act as a light-driven outward H^+^-pump or an inward Cl^−^ ion pump, respectively (Figure 1). BR and HR contribute to the formation of a membrane potential and thus function in light-energy conversion. The two other *H. salinarum* rhodopsins are sensory rhodopsin I and II (SRI and SRII) (Spudich and Bogomolni, 1984; Jung et al., 2003), which act as positive and negative phototaxis sensors, respectively, by activating transmembrane transducers (Figure 1). For the first 30 years since the early 1970's, microbial rhodopsins were epitomized by haloarchaeal proteins, the first-discovered and best-studied light-driven H^+^ pump BR and its close relatives (HR, SRI, and SRII). However, over the past 15 years, so many related photoactive proteins with similar or different functions were identified in *Archaea, Eubacteria*, and *Eukaryota*, and are now collectively called microbial rhodopsins (Brown, 2013; Grote et al., 2014). Channelrhodopsin (ChR), a microbial rhodopsin found in green algae, functions as a light-gated cation channel (Nagel et al., 2002, 2003) (Figure 1). Discovery of ChR led to the emergence of optogenetics (Miesenböck, 2011), in which light-gated ion channels and light-driven ion pumps are used to depolarize and hyperpolarize selected cells of neuronal networks, respectively. There are high expectations that this new method can be used to understand the circuitry of the brain (Deisseroth, 2011; Diester et al., 2011).

In optogenetics, animal brain functions are studied by incorporating microbial rhodopsins, but not animal rhodopsins, into the animal brain. There are two reasons for this. One is the isomeric structure of the chromophore (Figure 2). An 11-*cis* retinal, the chromophore molecule of animal rhodopsins, is not generally abundant in animal cells. In contrast, endogenous all-*trans* retinal, the chromophore molecule of microbial rhodopsins, is sufficient for optogenetics in animal cells, and there is no need to add the chromophore. The second reason is “bleaching.” Upon light absorption, animal and microbial rhodopsins exhibit retinal isomerization from the 11-*cis* to all-*trans*, and all-*trans* to 13-*cis* forms, respectively (Ernst et al., 2014). Such an isomerization reaction is common, but the end of the photoreaction differs between animal and microbial rhodopsins. The isomerized all-*trans* retinal does not return to the 11-*cis* form in animal rhodopsins, and is thus called “photobleaching.” This is not a problem in human visual cells because enzymatically isomerized 11-*cis* retinal is newly supplied, but this is not the case in other animal cells. In contrast, the 13-*cis* form is thermally isomerized into the all-*trans* form, and the spontaneous return is termed the “photocycle” in microbial rhodopsins. Due to the existence of naturally abundant all-*trans* retinal and its photocycle feature, microbial rhodopsins have become a tool in optogenetics.

In optogenetics, light-gated ion channels and light-driven ion pumps are used to depolarize and hyperpolarize selected cells of neuronal networks (Boyden et al., 2005; Zhang et al., 2007; Chow et al., 2010). It is interesting that two different ion-transporting functions, channel and pump, take place in microbial rhodopsins, even though their structures are similar. In pumps, the transport pathways between the two sides of the membrane cannot be fully connected because the gradient formed by active transport will collapse. This is an important aspect when distinguishing pumps from channels. The former needs energy input, which ensures the uni-directionality of transport across the membrane. To achieve this, alternative access for both sides and a switch in pumps, which are controlled spatially and temporally, is considered to take place. In contrast, a channel needs a fully connected ion pathway for passive transport of ions upon opening.

In this paper, I review recent understanding of light-driven ion-pumping rhodopsins, which are expected to be used as neural silencers in optogenetics. A light-driven H^+^ pump was the first microbial rhodopsin discovered (BR), and metagenomic research identified thousands of new microbial rhodopsins from marine bacteria. The new rhodopsin is called proteorhodopsin (PR), and it is estimated that 50% of microbes in the photic zone possess PR genes (Béjà et al., 2000; de la Torre et al., 2003; Venter et al., 2004; Finkel et al., 2013). In addition to many PRs (H^+^ pump), light-driven inward Cl^−^ pumps that differ from HR have recently been reported (Inoue et al., 2014; Yoshizawa et al., 2014). In contrast to light-driven outward H^+^ and inward Cl^−^ pumps, no cation pumps are known, except for the H^+^ pump. This is a reasonable possibility, as retinal chromophore is positively charged in rhodopsins, and thus non-H^+^ cation-pumping rhodopsins are impossible because of electrostatic repulsion. However, an outward Na^+^ pump has already been naturally created in the ocean (Inoue et al., 2013), and can be used as a novel neural silencer.

**Figure 3:**
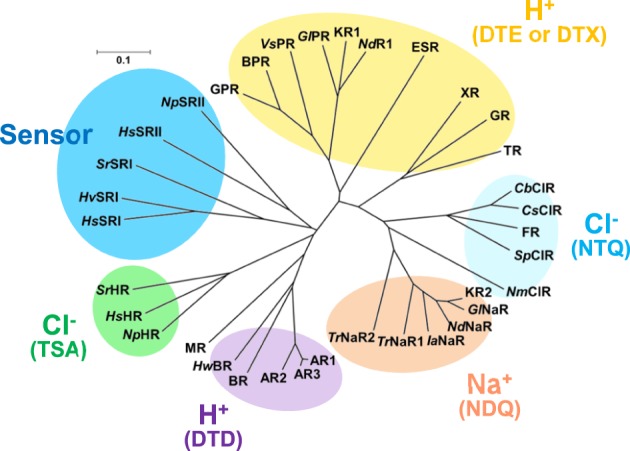
**Phylogenic tree of microbial rhodopsins**. This figure is modified from Inoue et al. (2015). The scale bar represents the number of substitutions per site (0.1 indicates 10 nucleotides substitutions per 100 nucleotides). Marine bacterial H^+^ (yellow), Na^+^ (orange) and Cl^−^ (cyan) pumps have the DTE (or DTX), NDQ, and NTQ motifs, respectively, while archaeal H^+^ and Cl^−^ pumps have the DTD and TSA motifs, respectively. Sensory rhodopsins from halophilic archaea and eubacteria are also shown. AR1, Archaerhodopsin-1; AR2, Archaerhodopsin-2; AR3, Archaerhodopsin-3; *Hw*BR, BR from *Haloquadratum walsbyi*; MR, Middle rhodopsin; *Np*HR, *Hs*HR, *Sr*HR, HR from *Natronomonas pharaonis*; *H. salinarum* and *Salinibacter ruber*; *Hs*SRI, *Hv*SRI, *Sr*SRI, sensory rhodopsin I from *H. salinarum, Haloarcula vallismortis* and *S. ruber*; *Hs*SRI, *Np*SRI, sensory rhodopsin I from *H. salinarum* and *N. pharaonis*; *Vs*PR, *Gl*PR, *Nd*R1. proteorhodopsins from *Vibrio* sp. AND4, *Gillisia limnaea* DSM 15749, *Nonlabens dokdonensis* DSW-6; XR, xanthorhodopsin, TR, proteorhodopsin from *Thermus thermophilus*; *Cb*ClR, *Cs*ClR, *Sp*ClR, NmClR, ClR from *Citromicrobium bathyomarinum, Citromicrobium* sp. JLT1363, *Sphingopyxis baekryungensis* DSM 16222 and *N. marinus*; *Gl*NaR, *Nd*NaR, *Ia*NaR, *Tr*NaR1, *Tr*NaR2, NaR from *G. limnaea, Nonlabens dokdonensis, Indibacter alkaliphilus*, and two NaRs from *Truepera radiovictrix*, respectively.

**Figure 4:**
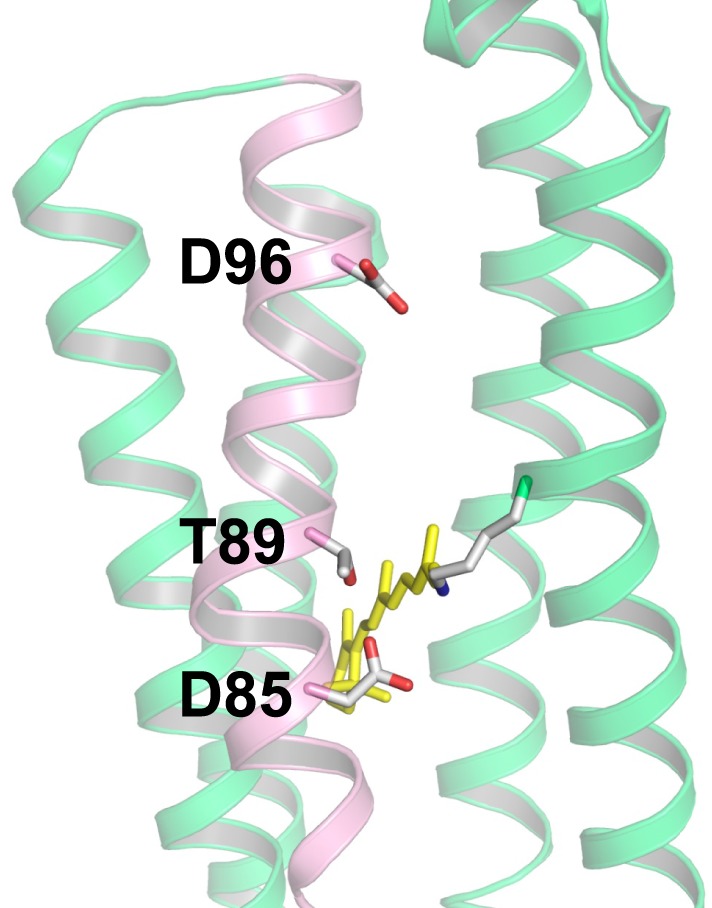
**Structure of bacteriorhodopsin (BR) with the DTD motif (PDB: 1QM8, Takeda et al., 1998)**. The three amino acid residues of the motif (D85, T89, and D96) are located in the C-helix (pink), while other helices are shown in green. Among the seven helices, the A-helix and B-helix are removed to provide a clear view. In BR, D85, and D96 act as the H^+^ acceptor and donor to the Schiff base, respectively, and T89 forms a hydrogen bond with D85.

We now know that nature uses three different ion pump rhodopsins (H^+^, Na^+^, and Cl^−^ pumps). They can be distinguished by characteristic sequences (Figure 3). BR has two aspartic acid residues, D85 and D96, in helix C which function as the H^+^ acceptor and donor, respectively, for the retinal Schiff base during its H^+^ pumping photocycle (Figure 4). In addition, the former forms a hydrogen bond with T89. The DTD motif in BR (D85, T89, and D96) is well conserved for other archaeal H^+^-pumping rhodopsins. At the corresponding position, most PRs have a DTE motif in which the H^+^ donor is Glu instead of D96 in BR. However, there are some exceptions. *Exiguobacterium sibiricum* rhodopsin (ESR) has Lys instead of Glu (Petrovskaya et al., 2010), and the DTX motif may be more accurate for the marine bacterial H^+^ pump. Light-driven Cl^−^ pump HR is a TSA rhodopsin, and light-driven Na^+^ and Cl^−^ pumps have NDQ and NTQ motifs, respectively at the same position (Figure 3) (Inoue et al., 2013; Béjà and Lanyi, 2014; Yoshizawa et al., 2014). Thus, these three residues, which correspond to position 85, 89, and 96 of BR, are important for categorizing ion pump rhodopsins. The next section summarizes structural features of these light-driven pumps, followed by a mechanistic explanation on H^+^, Cl^−^, and Na^+^ pumps, one by one.

## Structural features of ion-pumping microbial rhodopsins

**Figure 5:**
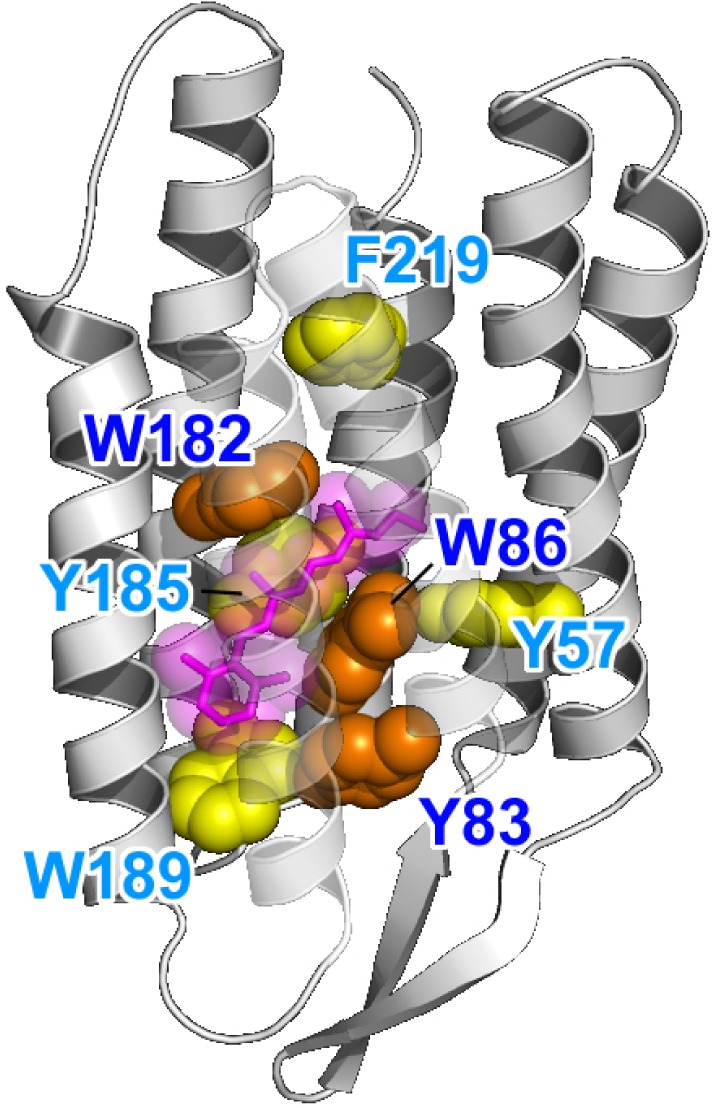
**Highlighted BR structure with the retinal chromophore, W86, W182, and Y185 (PDB: 1QM8)**. Y83, W86, and W182 are strongly conserved among the microbial rhodopsins (orange). Aromatic residues are strongly conserved at the Y185, W189, and F219 positions (yellow). In BR, W86, W182, Y185, and W189 constitute the chromophore binding pocket for all-*trans* retinal (red).

**Figure 6:**
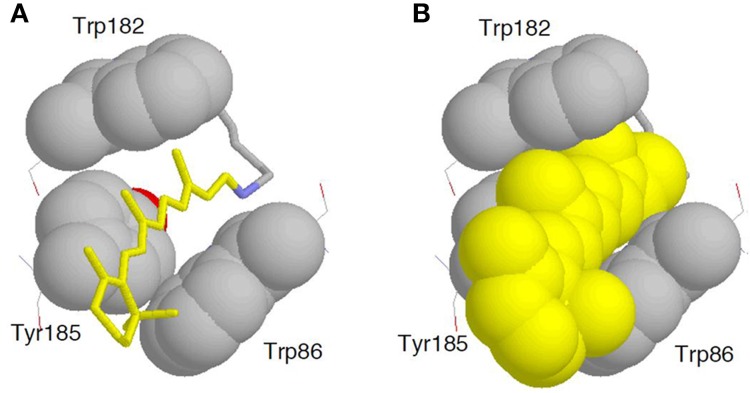
**Enlarged structure of BR with the retinal chromophore, W86, W182, and Y185 (PDB: 1QM8)**. All-*trans* retinal (**A**, yellow stick drawing; **B**, yellow space-filling drawing) is embedded in the binding pocket comprised of these aromatic amino acids.

Despite a variety of sequences and functions, the structural and mechanistic principles of microbial rhodopsin architecture have one common structure, a tight alpha-helical bundle of seven transmembrane helices surrounding the retinal chromophore (Klare et al., 2008; Zhang et al., 2011; Brown, 2013; Ernst et al., 2014). Figure 5 illustrates the overall structure of BR, which highlights the conserved aromatic amino acids. The retinal binding pocket is the most conserved element of the structure. W86, W182, and Y185, which constitute an important part of the chromophore binding site, are perfectly conserved among ion-pumping microbial rhodopsins (Figure 6). The presence of these bulky groups possibly determines the isomerization pathway from the all-*trans* to the 13*-cis* form after light absorption. Moreover, the interaction of photoisomerized retinal with W182 may serve as a mechanical transducer for passing the energy stored in retinal deformation into functionally important changes of the helical tilts necessary for function (Luecke et al., 2000). Y185 in BR (Figure 6) participates in hydrogen-bonding stabilization of the Schiff base counterion for many rhodopsins.

**Figure 7:**
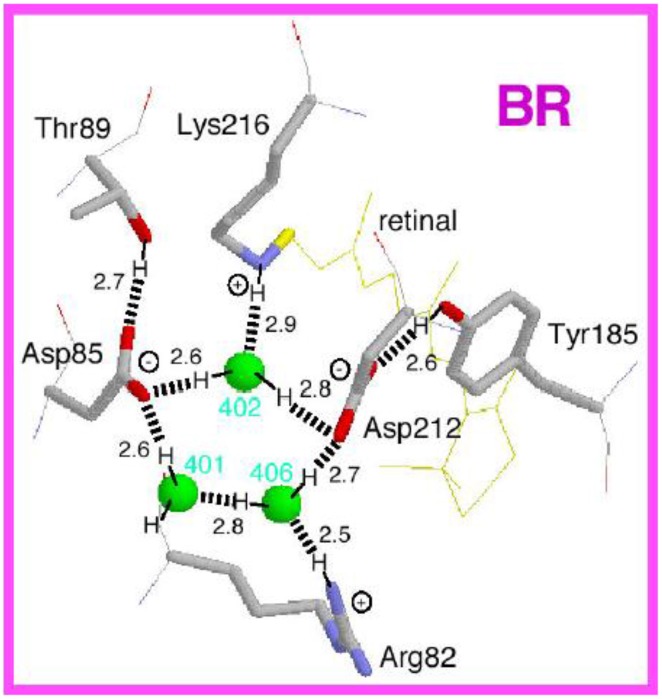
**Structure of the Schiff base region in bacteriorhodopsin (BR)**. This is the side view of the Protein Data Bank structure 1C3W, which has a resolution of 1.55 Å (Luecke et al., 1999). The membrane normal is approximately in the vertical direction of this figure. Hydrogen atoms and hydrogen bonds (dashed lines) are supposed from the structure, while the numbers indicate hydrogen-bonding distances in Å.

In addition to the aromatic sidechain rings, electrostatic and hydrogen-bonding interactions in the proximal region of retinal are crucial in defining the functionality of microbial rhodopsins (Ernst et al., 2014). The sidechain of K216 in BR (or its homologs in other microbial rhodopsins) forms a covalent linkage with the retinal molecule through the Schiff base (Figure 7). As the Schiff base is usually protonated, K216 and super-conserved R82 of helix C in BR provide two positive charges within the protein (Figure 7), which requires two negative charges for electrostatic stabilization. This dictates the most common configuration of the Schiff base counterion, which includes two perfectly conserved carboxylic acids (D85 and D212 in BR) for H^+^-pumping microbial rhodopsins (Figure 7).

Other functions in microbial rhodopsins originate from deviation from this arrangement. For example, the negatively charged Asp at position 85 is replaced by Thr in Cl^−^-pumping HR, which requires a negative charge for electrostatic stabilization. This is the driving force of Cl^−^ binding near the retinal chromophore in Cl^−^ pumps. In ChR, the corresponding amino acid of D85 is Glu, and Tyr at position 185 of BR is replaced by Phe, which is possibly linked to the channel function. Ii should be emphasized that all microbial rhodopsins contain protein-bound water molecules near the Schiff base (Figure 7), probably contributing to the stabilization of the protonated Schiff base in the hydrophobic protein interior (Kandori, 2000; Heberle, 2004; Wolf et al., 2008). These water molecules play a key role in protein function, and have been extensively studied by X-ray crystallography of photointermediates, Fourier transform infrared spectroscopy (FTIR) spectroscopy and computational methods (Gerwert et al., 2014). The electrostatic quadrupole in the Schiff base region is characteristic of most microbial rhodopsins, and light-induced retinal isomerization causes a hydrogen-bonding alteration of this region as well as steric effects, leading to various functions of microbial rhodopsins.

## Light-driven proton pumps

BR from *H. salinarum* is the first discovered microbial rhodopsin (Oesterhelt and Stoeckenius, 1971) and the first membrane protein whose structure was found to be composed of seven helices by electron microscopy (Henderson and Unwin, 1975). BR is also the first membrane protein to have its amino acid sequence determined (Khorana et al., 1979). As the best studied microbial rhodopsin, it serves as a paradigm of a light-driven retinal-binding ion pump and aids in studies of novel rhodopsins. Archaerhodopsin 3 (Arch), the best used protein in optogenetics as a neural silencer (Chow et al., 2010), has a DTD motif, and its molecular mechanism is similar to that of BR (58% amino acid identity).

**Figure 8:**
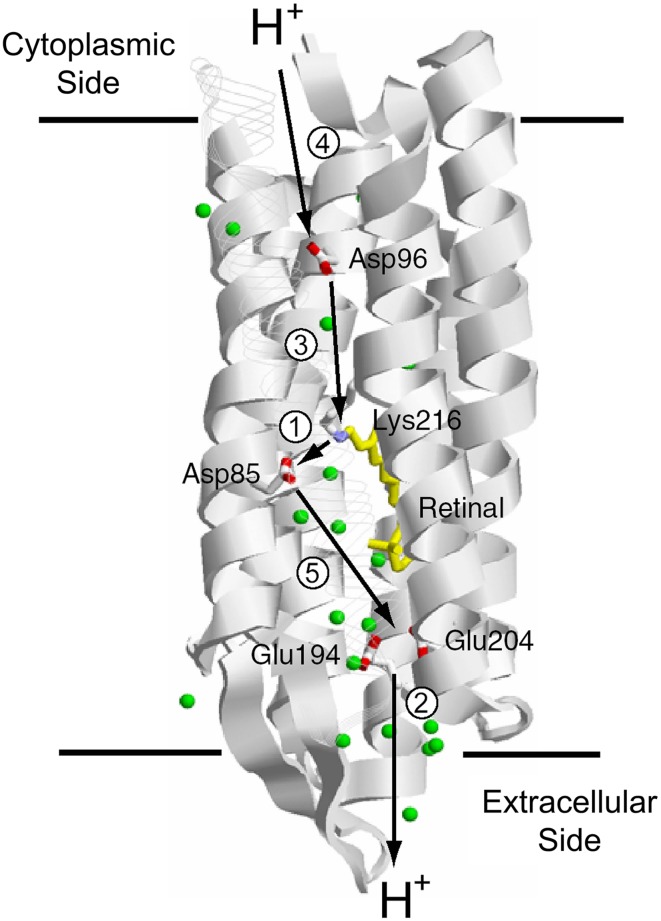
**H^+^ transport pathway in bacteriorhodopsin (BR)**. Arrows indicate each H^+^ transfer, and the numbers indicate a temporal order; (1) Schiff base to D85, (2) H^+^ release, (3) D96 to Schiff base, (4) uptake, and (5) D85 to the H^+^ release group.

**Figure 9:**
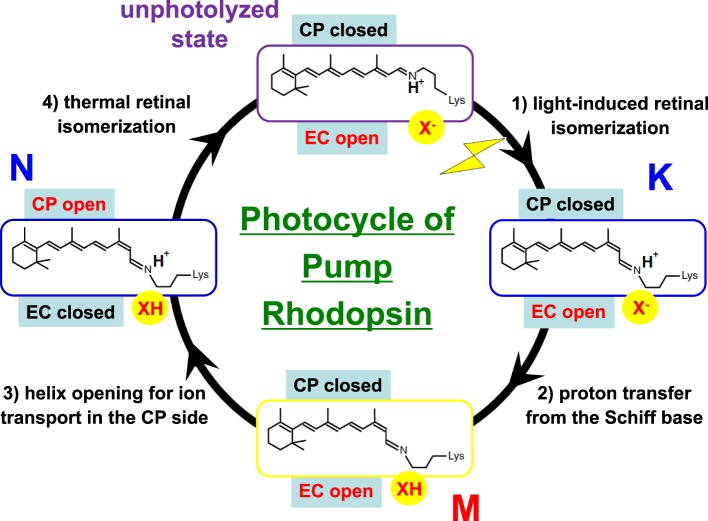
**Typical photocycle of microbial rhodopsins showing isomeric and protonation state of retinal**. X^−^ represents the Schiff base counterion, and D85 in BR also acts as the H^+^ acceptor from the Schiff base. In a Cl^−^ pump such as HR and FR, X^−^ is a Cl^−^, so that the M intermediate is not formed because the Schiff base is not deprotonated. Instead, the Cl^−^ is transported upwards (in this figure). In KR2, a Na^+^ pump, X^−^ is a D116 acting as the Schiff base counterion and H^+^ acceptor from the Schiff base. CP and EC indicate cytoplasmic and extracellular domains, respectively. In the unphotolyzed state of microbial rhodopsins, the EC side is generally open through a hydrogen-bonding network but the CP side is closed. While this is persistent in the K and M states, the CP side is open in the N state. When the EC side is closed (black), the CP side is open, as is the case for an ion pump, as occurs in the N intermediate of BR. Such alternative access must work for all H^+^, Cl^−^, and Na^+^ pumps.

The H^+^ pathway across the membrane from the cytoplasmic to the extracellular side in BR is shown in Figure 8, together with protonatable groups and the order of respective H^+^ transfers. A summary of the photocycle is shown in Figure 9, which illustrates key intermediate states for most microbial rhodopsins. Although the photocycle of BR contains six intermediates, namely J, K, L, M, N, and O states that are named alphabetically, only three states (K, M, and N) are shown in Figure 7 to demonstrate the mechanism clearly. After light absorption, photoisomerization occurs from the all-*trans*- to 13-*cis-*form in 10^−13^ second. This ultrafast retinal isomerization yields the formation of red-shifted J and K intermediates, in which J is the precursor of the K state. The protein cavity, which accommodates retinal, cannot change its shape promptly, and the K intermediate contains twisted 13-*cis* retinal. An altered hydrogen-bonding network in the Schiff base region also contributes to higher free energy in K than in the original state, and such energy storage in the primary intermediate structure leads to subsequent protein structural changes upon relaxation.

In the case of BR, relaxation of the K intermediate leads to the formation of the blue-shifted L intermediate. For H^+^-pumping rhodopsins, as well as some photosensory rhodopsins, the L intermediate serves as the precursor of the H^+^ transfer reaction from the Schiff base to its primary carboxylic H^+^ acceptor, by which the M intermediate is formed. This is a key step in H^+^ transport. Since the M intermediate has a deprotonated 13-*cis* chromophore, it exhibits a characteristically strong blue-shifted absorption (λ_max_ at 360–410 nm), and is well isolated from other intermediates. In BR, the H^+^ acceptor (X^−^ in Figure 9) is D85, so that the primary H^+^ transfer takes place from the Schiff base to D85. T89 in the DTD motif forms a hydrogen bond with D85, which is also persistent even after formation of the M intermediate (Kandori et al., 2001).

**Figure 10:**
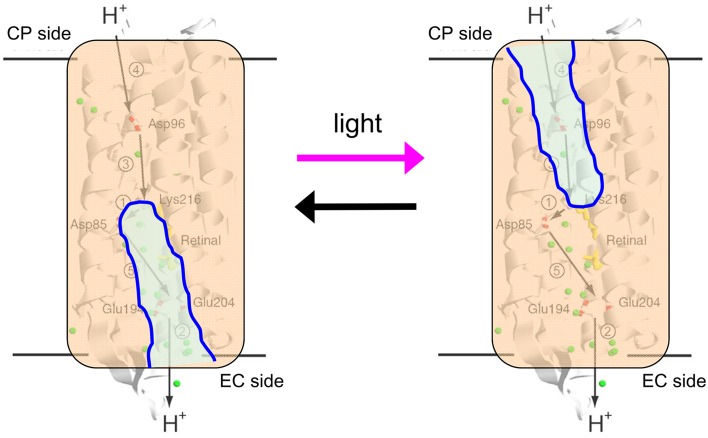
**Schematic drawing of alternative access in BR**.

If the Schiff base of M is reprotonated from D85 in BR (the first D in DTD), no H^+^ transport occurs. In reality, the Schiff base is reprotonated from D96 (the last D in DTD) in the cytoplasmic region (Figures 6, 8), by which the N intermediate is formed (Gerwert et al., 1989). The molecular mechanism of unidirectional transport of H^+^s in BR has attracted the attention of many researchers, and it is believed that the primary H^+^ transfer from the Schiff base to D85, and the subsequent H^+^ transfer from D96 to the Schiff base determine the unidirectionality from the cytoplasmic to the extracellular region. The crystal structure of BR exhibits an asymmetric pattern of hydration: while seven internal water molecules are found in the extracellular half, only two are observed in the cytoplasmic half (Figure 10). Such asymmetry makes sense in view of BR's function, as the water molecules build a hydrogen-bonding network on the extracellular side for fast H^+^ release while the cytoplasmic side is likely inaccessible in the dark and allows H^+^ uptake only after the light-induced accessibility switch. Such asymmetric access (EC open and CP closed) is not only the case for the unphotolyzed state, but is also the case for the K and M intermediates, as shown in Figure 9.

To make H^+^ conduction in the cytoplasmic region possible (CP open), an additional conformational alteration should occur to allow the entrance of water into the vicinity of D96. Such a conformational change is realized mainly by outward motion of the cytoplasmic half of helix F and the N intermediate is often characterized as the largest changes in the protein backbone conformation. Such helical motions are functionally significant both for ion transport and interactions with transducers of sensory rhodopsins (Klare et al., 2008; Spudich et al., 2014). In fact, this is also the case for animal rhodopsins (Scheerer et al., 2008; Rasmussen et al., 2011), and in addition, such helix opening is believed to be the general mechanism of activation for G-protein coupled receptors. The photocycle usually ends with another red-shifted intermediate, the O intermediate, resetting the original unphotolyzed conformation. Thermal isomerization, which distinguishes “photocycling” microbial rhodopsins from “bleaching” animal rhodopsins, takes place in the transition from N to O. Large conformational alterations in N possibly act as an isomerase in the transition from 13-*cis* to all-*trans*. Thus, light-induced retinal isomerization drives protein structural changes at the beginning, while protein drives thermal isomerization of retinal at the end.

The photocycle of PR, a rhodopsin of the DTE motif, has many similarities to that of BR (Inoue et al., 2015). Five intermediates, K, M_1_, M_2_, N, and PR′, have been identified in the photocycle of PR, where PR′ represents the transient intermediate with identical absorption spectra as the initial state. Unlike BR, no significant accumulation of the L intermediate is observed in the photocycle of PR, probably for kinetic reasons. Another difference with BR is the red-shifted absorption of N. The red-shifted intermediates should be termed O, analogous to BR, but time-resolved FTIR spectroscopy suggested that the late red-shifted intermediate of PR has a 13-*cis* form, similar to the N of BR (Dioumaev et al., 2002). It is noted that the isomeric form is explicitly identified using C_12_ and C_14_ deuterated retinal, as was reported previously (Curry et al., 1984). Some marine bacteria possess an H^+^ pump with the DTX motif, where X is neither Asp nor Glu, which are convenient amino acids to alter pKa, and are thus largely involved in the intramolecular H^+^ transfer of proteins. The molecular mechanism of H^+^ transfer for DTX rhodopsins such as ESR (DTK in this case) is intriguing (Balashov et al., 2013).

## Light-driven chloride ion pumps

**Figure 11:**
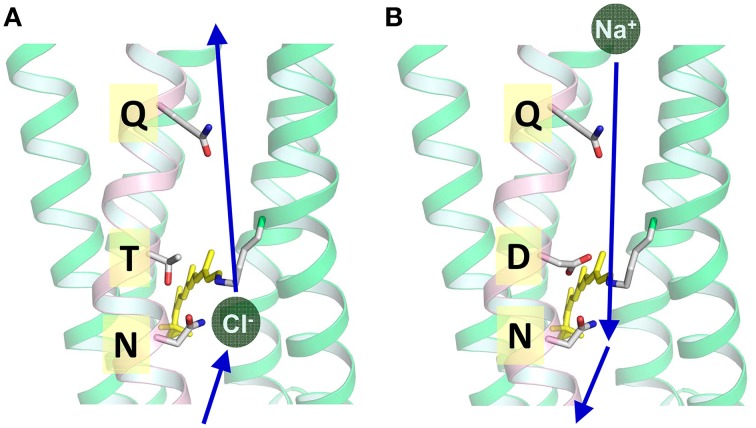
**(A)** Cl^−^ transport pathway in HR and FR (NTQ rhodopsin). Cl^−^ binds near the Schiff base region, as is seen from the Cl^−^-dependent color change. The KR2 structure is used in which T is replaced by D116. **(B)** Na^+^ transport pathway in KR2 (NDQ rhodopsin). Na^+^ does not bind near the chromophore in the dark, while a light-induced structural alteration accompanies the uptake of Na^+^ upon formation of the O intermediate. This is the structure of KR2 (PDB: 3X3C, Kato et al., 2015).

The first identified light-driven inward Cl^−^ pump was HR in 1977 (Matsuno-Yagi and Mukohata, 1977). Interestingly, it was first believed to be an outward Na^+^ pump, whereas clear anion dependence revealed that HR is an inward Cl^−^ pump (Schobert and Lanyi, 1982). While the overall architecture of HRs is BR-like, the crystal structures of two Cl^−^ pumps from *Halobacterium salinarum* (*Hs*HR; Kolbe et al., 2000) and from *Natronomonas pharaonis* (*Np*HR; Kouyama et al., 2010) clearly show the presence of a Cl^−^ in the Schiff base region. HR has the TSA motif in which D85 in BR is replaced by Thr. This suggests that the electric quadrupole of the Schiff base with its counterion complex (D85, D212, and R82 in BR) lacks a negative charge and that the charge balance is compensated for by the binding of the negatively charged Cl^−^. This is also the case for NTQ rhodopsins such as Fulvimarina rhodopsin (FR), where D85 is replaced by Asn (Figure 11A). FR clearly shows Cl^−^-dependent color changes, indicating direct binding near the Schiff base as well as HR (Inoue et al., 2014).

In HR, hydrogen bonds of the Schiff base and of protein-bound water molecules are weak (Shibata et al., 2005), suggesting that the transported Cl^−^ is not involved in strong hydrogen-bonding, in contrast to H^+^ transport in BR. After light absorption, photoisomerization occurs from the all-*trans* to the 13-*cis* form in an ultrafast timescale, yielding the formation of the primary K intermediate. Relaxation of the K intermediate in HR leads to the formation of the blue-shifted L intermediate. In BR, the primary H^+^ transfer takes place from the Schiff base to D85. In the case of Cl^−^ pump HR, the Schiff base does not deprotonate during the photocycle, because Asp85 in BR is replaced by Thr. In HR and FR, X^−^ in Figure 9 is a Cl^−^, and the accessibility of Cl^−^ in the unphotolyzed state must be the EC side, being supported by a CP closed structure of HR (Kolbe et al., 2000; Kouyama et al., 2010). During the photocycle, Cl^−^ is directly translocated upon decay of the L intermediate (Essen, 2002). Two L intermediates (L_1_ and L_2_, sometimes called L and N) were observed by various methods, suggesting an extracellular to intracellular change in accessibility during their interconversion, analogous to the M_1_ and M_2_ intermediates of BR (Ernst et al., 2014). Photoisomerization causes changes in the electric and hydrogen-bonding environment of the Cl^−^ binding site, which drives its movement to the cytoplasmic side of the protonated Schiff base (Shibata et al., 2005). To make Cl^−^ conduction in the cytoplasmic region possible, a conformational change is needed on the CP side (CP open) while the EC side should be closed when the Cl^−^ conduction channel is open on the cytoplasmic side. The hydrogen bond of the Schiff base is strengthened in the L intermediate, but the hydrogen-bonding acceptor of the Schiff base is not Cl^−^. It is most likely a water molecule. Water-containing hydrogen-bonding network is rearranged, which probably opens the valve to the cytoplasmic region, and Cl^−^ is released to the cytoplasmic side during the transition to the O intermediate (Gruia et al., 2005; Kanada et al., 2011).

Interestingly, BR can be converted into an HR-like Cl^−^ pump by a single D-to-T amino acid replacement at D85 (Sasaki et al., 1995; Tittor et al., 1997). This suggests that the amino acid at position 85 being a determinant for ion specificity. Nevertheless, the reverse T-to-D mutations of HR, such as T108D of *Hs*HR and T126D of *Np*HR, does not convert HR into a BR-like outward H^+^ pump (Havelka et al., 1995; Váró et al., 1996). These observations may imply that the molecular determinants of an H^+^ pump are more demanding than those of a Cl^−^ pump. Indeed, *Np*HR mutated to contain 10 key BR-like amino acids but lacking strongly hydrogen-bonded water, the functional determinant of the H^+^ pump (Muroda et al., 2012). Although it is not easy by mutation, HR can be converted into an H^+^ pump by the simple addition of sodium azide. Azide probably serves as an artificial H^+^ shuttle, suggesting common elements in the transport mechanism of H^+^ and Cl^−^ pumps (Hegemann et al., 1985; Váró et al., 1996). The restoration of strongly hydrogen-bonded water for the azide-bound HR is completely consistent with these results (Muneda et al., 2006).

About 15 years ago, an interesting hypothesis was proposed that BR might not be an outward H^+^ pump, but instead an inward OH^−^ pump. The idea was gained from intermediate structures of BR and a Cl^−^-pumping BR mutant (D85S) (Luecke, 2000; Facciotti et al., 2004), which raised an important question about the H^+^ pump. In the case of Na^+^ and Cl^−^ pumps, proteins transport Na^+^ and Cl^−^, respectively. What then does the H^+^ pump transport? An outward H^+^ pump can be achieved by transporting (i) H^+^ outwardly, (ii) H_3_O^+^ outwardly, or (iii) OH^−^ inwardly. The easy conversion of BR into a Cl^−^ pump by a point mutation was interpreted as supporting evidence of BR being an inward OH^−^ pump. However, H^+^ transport is also possible by (iv) the Grotthuss mechanism (concerted H^+^ transfer through water chains) (Agmon, 1995), and this most likely takes place in light-driven H^+^ pumping rhodopsins (Freier et al., 2011). It should be noted that the hypothesis of BR as an inward OH^−^ pump has been never denied experimentally.

A spectroscopic study of FR, an NTQ rhodopsin, revealed a surprising similarity between two different Cl^−^ pumps of NTQ and TSA rhodopsins, even though they are evolutionarily distant (Inoue et al., 2014). A common mechanism of binding and transport of Cl^−^s suggests the importance of the local structure in the Schiff base region. In an NTQ rhodopsin like FR, two positive charges and a negative charge (R82, protonated Schiff base and D212 in BR) are conserved, and binding of Cl^−^ satisfies the charge balance near the Schiff base.

## Light-driven sodium ion pumps

For more than 40 years after the first report of BR, Na^+^-pumping rhodopsin was not discovered. This absence was thought to likely be because the protonated Schiff base, a positive charge, exists on the ion conductive pathway and must inhibit the transport of non-H^+^ cations. However, a light-driven Na^+^ pump rhodopsin (NaR) was found in the flavobacterium *K. eikastus* (Inoue et al., 2013). *K. eikastus* has two rhodopsin genes (KR1 and KR2). The former has a typical PR-like sequence with a DTE-motif, and shows an outward H^+^ pump function. On the other hand, KR2, possessing the NDQ motif, was shown to be a new outward Na^+^ pump rhodopsin, based on its light-induced alkalization of a cell suspension. Since the residues in helix C form an ion conduction pathway, three residues in the NDQ motif are important for the transport of Na^+^, as was revealed by a mutation study (Inoue et al., 2013). More than 10 rhodopsin genes with an NDQ-motif have been identified to date, indicating that Na^+^ pump rhodopsins are diversely used among various species in nature (Figure 3).

Light absorption generates the red-shifted K-intermediate with 13-*cis* retinal (Ono et al., 2014), leading to the photocyclic reaction which recovers to the initial state in 100 ms. Following the K-intermediate, the L ⇄ M and O intermediates appear in this order of accumulation (Inoue et al., 2013; Balashov et al., 2014). The nomenclature of these intermediates is according to that of BR based on their absorption spectra and the time-scale in which each intermediate appears. While the K and O intermediates have red-shifted absorption, the spectrum of L ⇄ M is blue-shifted compared with the initial state. In particular, M (λ_max_ = 400 nm) has a more than 100-nm shorter absorption wavelength than KR2 (λ_max_ = 526 nm), as the retinal Schiff based is deprotonated in this state. In the D116N mutant, the M intermediate does not accumulate, supporting the notion that the H^+^ acceptor of KR2 is D116, making up an NDQ-motif. The rate of O-accumulation is accelerated by an increase in Na^+^ concentration, suggesting that Na^+^ uptake occurs in the L ⇄ M-to-O process.

**Figure 12:**
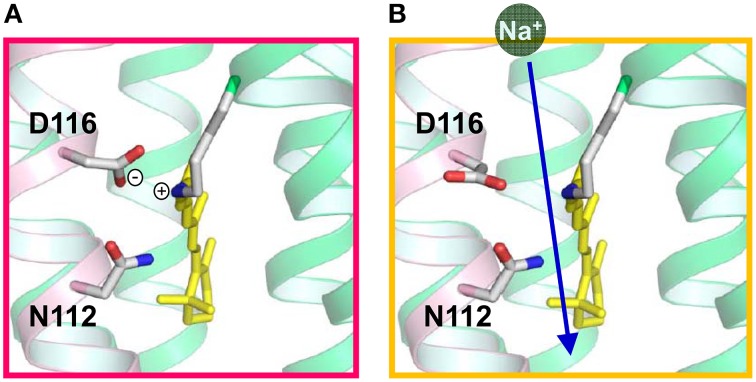
**Proposed local structures of KR2 in the dark (A) and in the M intermediate (B), suggested from crystal structures of KR2 at different pHs**.

These findings explain how Na^+^ is transported to the extracellular region across the protonated Schiff base of retinal. In the case of H^+^ transport by H^+^ pump rhodopsin, the H^+^ bound to the Schiff base itself is transported. In contrast, the transfer of H^+^ to the counterion (D116) upon M formation of KR2 transiently eliminates the positive charge in the Schiff base region, enabling the conduction of Na^+^ beside the Schiff base during the (L ⇄ M)-to-O process (Figure 11B). This scenario is strongly supported by recent crystal structures of KR2, which were determined at acidic and neutral pHs (Kato et al., 2015). Although there were few structural differences at different pHs, a change in the orientation of D116 was observed (Figure 12). Therefore, it is likely that D116 interacts with the protonated Schiff base in the unphotolyzed state, while protonated D116 newly interacts with N112 (and S70), allowing Na^+^ uptake from the cytoplasmic side by electrostatic neutralization (Kato et al., 2015). Upon formation of the red-shifted O intermediate, the Schiff base gains H^+^ again presumably from D116, and the ion-pair between the Schiff base and D116 inhibits the backward flow of transported Na^+^.

KR2 is a light-driven Na^+^ pump that can also pump Li^+^. On the other hand, KR2 pumps H^+^ in K^+^, Rb^+^, and Cs^+^. Thus, KR2 is a compatible Na^+^-H^+^ pump, although it functions as a light-driven Na^+^ pump under physiological conditions in the ocean. A light-driven Na^+^ pump from *Gillisia limnaea* does not pump H^+^ (Balashov et al., 2014), suggesting a variety of Na^+^ pumping rhodopsins. A mutation study revealed important residues for each pump function (Inoue et al., 2013). Both Na^+^ and H^+^ pump functions were completely lost for R109A, D251A, D251N, and D251E, indicating the important role of R109 and D251, which correspond to R82 and D212, respectively, in BR (Figure 7). Both functions were lost for D116A and D116N, while D116E pumps only H^+^. Similarly, N112A pumps only H^+^, though N112D pumps both Na^+^ and H^+^. D116 and N112 in the NDQ motif correspond to T89 and D85, respectively, in BR (Figures 4, 7, 11B, 12). S70A pumps only H^+^, while S70T pumps both Na^+^ and H^+^. These results demonstrate the importance of charged residues such as R109, D116, and D251, and the hydrogen-bonding interaction involving S70 and N112. The lack of a Na^+^ pump for S70A, N112A, and D116E may originate from a narrowed Na^+^ pathway by mutation and/or less stabilization of Na^+^ binding in the O intermediate.

H^+^ and Cl^−^ pump rhodopsins bind the substrate ions near the active center in the dark. In the case of H^+^ pumps (BR, PR, etc.), substrate H^+^ is bound to the retinal Schiff base and acidic amino acid residues constitute the H^+^-transfer pathway. The binding site of Cl^−^ in HR was identified by X-ray crystallography in the vicinity of the protonated Schiff base. While the binding of ions affects the colors of H^+^ pumps and HRs, KR2 does not show any change in color between the presence and absence of Na^+^ (Inoue et al., 2013). This implies that the Na^+^ binding site of KR2 is distant from retinal. Thus, the binding of Na^+^ to KR2 cannot be studied by conventional UV-visible absorption spectroscopy. However, a conformational change of KR2 upon Na^+^-binding (*K*_*d*_ of 11.4 mM) was clearly observed by using attenuated total reflection-Fourier transform infrared (ATR-FTIR) spectroscopy, and a mutation study showed that Na^+^ binds to the extracellular surface (Inoue et al., 2013). The Na^+^ binding site was directly recently visualized by its crystal structure (Gushchin et al., 2015). It is intriguing that several mutants do not bind Na^+^ but can transport Na^+^, indicating that Na^+^ binding is not a prerequisite for pump function (Inoue et al., 2013). The role of Na^+^ binding to KR2 is likely to increase thermal stability of the protein (Gushchin et al., 2015; Kato et al., 2015).

The H^+^ pump mechanism by KR2 is the least understood to date. The efficiency of the H^+^ pump is much lower than that of other H^+^ pumps, and the H^+^ pumping photocycle is >10 times slower than the Na^+^ pumping photocycle (Inoue et al., 2013). As KR2 functions as a Na^+^ pump in the ocean, it is plausible that the H^+^ pump is never important for KR2. It should be noted, however, that the concentration of Na^+^ in the ocean is 0.4 M and that of H^+^ is 10^−8^ M (pH 8), a difference of more than seven orders of magnitude. Therefore, Na^+^ and H^+^ transport needs to be compared under similar conditions for a better understanding of the mechanism. In the absence of Na^+^ and Li^+^ in the intracellular medium, it is likely that H^+^ is taken up from the cytoplasmic side to the Schiff base, when the Schiff base proton is transferred to D116. Then, H^+^ attached to D116 is released to the extracellular aqueous phase. This is a reasonable hypothesis based on current knowledge of natural H^+^ pumps. However, the molecular mechanism of the H^+^ pump by KR2 needs to be examined in detail.

## Perspectives

This review provides a recent understanding of ion-pumping rhodopsins. Our knowledge has changed dramatically following the emergence of new classes of microbial rhodopsins. As the metagenomic analysis of the ocean environment is still ongoing, newly identifying rhodopsin genes, it is expected that additional functional rhodopsins will still be found (Venter et al., 2004; Finkel et al., 2013). As H^+^ and Cl^−^ pump rhodopsins are now used as optogenetic tools for optical silencing of neurons (Zhang et al., 2007; Chow et al., 2010), it can be expected that new rhodopsins from marine bacteria will provide a molecular basis for developing novel optogenetic tools. In particular, Na^+^ pump rhodopsin would be a potentially useful tool (Kato et al., 2015), because its outward Na^+^-transport evokes hyperpolarization of membrane potential without unphysiological intracellular pH change and Cl^−^ accumulation. In addition, based on the structure of KR2, a light-driven K^+^ pump has been created (Gushchin et al., 2015; Kato et al., 2015). This fact clearly shows that an understanding of the structure-function relationship in microbial rhodopsins is a prerequisite for novel molecular design.

### Conflict of interest statement

The author declares that the research was conducted in the absence of any commercial or financial relationships that could be construed as a potential conflict of interest.
